# Effect of a MAST Exercise Program on Anthropometric Parameters, Physical Fitness, and Serum Lipid Levels in Obese Postmenopausal Women

**DOI:** 10.2478/hukin-2014-0069

**Published:** 2014-10-10

**Authors:** Bartosz Trabka, Igor Z. Zubrzycki, Zbigniew Ossowski, Olgierd Bojke, Anna Clarke, Magdalena Wiacek, Ewelina Latosik

**Affiliations:** 1 Jędrzej Śniadecki Academy of Physical Education and Sport, Gdańsk, Poland.; 2 Department of Microbiology and Biochemistry, University of Fort Hare, Alice, South Africa.; 3 Department of Theory of Sports Training, Academy of Physical Education in Katowice, Poland.

**Keywords:** exercise, postmenopause, physical fitness, obesity, serum lipids, nordic-walking

## Abstract

The purpose of this study was to examine an influence of a mixed aerobic and strength training program (MAST) on anthropometry, serum lipid levels, physical performance, and functional fitness in obese postmenopausal women. The MAST sessions were held three times per week, and the exercise program lasted for 10 weeks. The exercise group demonstrated a statistically significant improvement in maximal oxygen uptake, a waist/hip ratio, and strength of the upper and lower body. An increase in LDL-C levels was observed in the control group. A 10-week MAST program encompassing Nordic-walking as an aerobic component, and strength exercises, induces positive changes in functional fitness, HDL-C, LDL-C and a waist/hip ratio in obese postmenopausal women. The observed changes implicate an increase in a health-related quality of life among the women administered to the physical exercise program.

## Introduction

Obesity is accompanied by an elevated Body Mass Index (BMI) and adverse lipid profiles. An interplay of these two factors may result in an increase of risk of cardiovascular diseases (Gorodeski, 2002) stemming into a decrease in Health-Related Quality of Life (HRQoL) (Fontaine and Barofsky, 2001). Although, obesity does not influence maximal aerobic capacity (Goran et al., 2000), it may decrease the level of physical fitness (Uthman and Aremu, 2007), and influence HRQoL (Hakkinen et al., 2010).

It has been shown that aerobic and strength exercises have a positive effect not only on levels of BMI and WC (Martins et al., 2010), but also on serum TC (Boyden et al., 1993; Lennon et al., 1983), TG (Kemmler et al., 2004a) and LDL-C levels (Hagner et al., 2009) and may augment a level of functional fitness among osteopenic postmenopausal women (Bravo et al., 1996).

Notwithstanding the plethora of information on correlations between physical exercises and menopause associated changes in physical fitness, functional fitness, serum lipid levels and maximal aerobic capacity, we still do not know what exercise intensity and/or what type of exercises is required to alleviate these detrimental phenomena in obese postmenopausal women.

The purpose of this pilot study was to determine the potential effect of a supervised mixed aerobic (Nordic-walking, NW) and strength training (MAST) program on anthropometry, serum lipids, physical performance expressed in terms of VO2max, and functional fitness in postmenopausal obese women.

## Material and Methods

### Participants

We conducted a 10-week randomized controlled trial with a parallel-group design of obese postmenopausal women. The Bioethics Commission of Regional Medical Chamber approved the study protocol, and all the participants gave their written informed consent.

In this study, the following inclusion criteria were employed: (a) postmenopausal female, i.e., the female who had the last period >12 months ago, and (b) BMI ≥ 25 kg/m^2^ and ≤ 40 kg/m^2^. Exclusion criteria were: (a) systolic BP ≥ 140 mmHg and/od diastolic BP ≥ 90 mmHg, (b) oophorectomy, (c) chemotherapy within six months before screening, (d) coronary artery disease, (e) renal failure, (f) rheumatoid arthritis, (g) pulmonary disease, (h) diabetes mellitus or type II diabetes treated with insulin, (i) myocardial infarction or surgery within six months before screening, (j) smoking of more than two cigarettes per week or consuming more than the equivalent of one glass of wine per day, and (j) inability to obtain approval for participation in the study from a primary-care physician.

**Figure 1:**
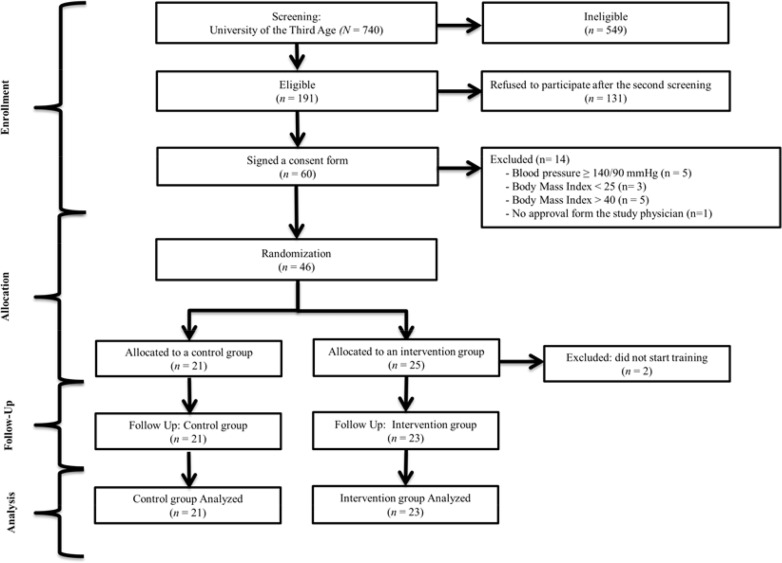
Flow chart of the study arrangement

All participants were recruited from the students attending a variety of lectures at the University of the Third Age. Of 740 women who agreed for the primary screening only 46 met all the criteria to participate in the study (Figure 1). The control and the intervention groups were randomly allocated through ballot drawing. Such an approach resulted in equal division of subjects into 2 groups comprising 23 subjects each. However, two patients refused to be included in the control group and requested to be included in the intervention group. This resulted in the control group comprising n = 21 subjects and intervention group n = 25 subjects. Notwithstanding a written agreement to participate in the study two patients from the intervention group were excluded due to personal reasons. Since all the subjects were allocated randomly to the intervention and the control group, we expected some statistical differences between both groups at baseline evaluations.

### Measures

#### Data Acquisition

The body height in cm was measured with accuracy of 0.1 cm. The subject was placed during measurement barefoot in the orthostatic position. Body mass in kg with accuracy of 0.1 kg was measured for each subject with minimal clothing. The body mass index (BMI) was calculated using the equation: body mass/height^2^ (kg/m^2^). Waist circumference (WC) was measured at the midpoint between the iliac crest and the external face of the last rib with accuracy of 0.1 cm. Hip circumference (HC) was measured as the maximal circumference over the buttocks with accuracy of 0.1 cm.

Functional fitness was assessed by a means of the Fullerton battery (Jones and Rikli, 2000). The battery comprised of the following tests: a) chair stand (CST), b) arm curl (CURL), c) chair sit and reach (CSR), and d) back scratch (UBF). The chair stand tests comprised of a number of full stands completed in 30 s. The arm curl employed the number of biceps curls completed in 30 s using a dumbbell of 2 kg. In the chair sit and reach test, the subject began in a sitting position with legs extended, and then attempted to reach their toes. In the flexibility test, the number of centimeters between the extended fingers, and toes was measured as a score. The back scratch test was performed with one hand reaching over the shoulder and other hand reaching around and up the back. The score was the number of centimeters between the extended fingers of both hands. All the tests were performed between breakfast and lunch in order to assess functional fitness and to avoid skewing of results by daily activities.

VO_2max_ peak was assessed by open-circuit spirometry using a modified protocol proposed by Ainsworth et al. (1997). In brief, (a) the test was preceded by a 2 min warm-up: walking at the speed of 2-3 km/hr, (b) the subject started walking, without holding onto the handrails during the test, at 4 km/hr and 0% grade. An elevation was increased by 2% each 2 min stage until volitional fatigue. The test was continued until the subject could not longer continue due to a too great elevation, achieved a respiratory-exchange ratio > 1.0, a maximal hear rate greater than HR = 206 – 0.88*age, or other clinical reason for test termination was observed. The heart rate, BP, and rating of perceived exertion using the Borg Scale (Borg, 1982) were obtained before the treadmill test, during the last 30 s of each stage and during a 6 min recovery period. VO_2max_ was measured using a breath-by-breath gas-exchange system (MetaMax 3B, Cortex-Medical, Germany). In this study, oxygen uptake data are expressed as VO_2_ peak.

#### Diet

Both the intervention group and the control group were preconditioned for 3 weeks before starting the experiment and the following dietary guidelines through the time of the experiment were employed: (a) no high-glycemic-index food (GI > 75), (b) no high-fat food, (c) no eating after 7 p.m. or three hours before sleep, (d) drinking at least 1.5 l of water per day, (e) eating five times a day, and (f) no drinking of alcohol during the test period. Each subject was asked to cover a total between 120–150% of the Basal Metabolic Rate (BMR)/day, based on the provided nutritive value of foods table (Gebhardt et al., 2002). All subjects were asked to make notes of their daily diet during the study period. To satisfy the experimental demands weekly briefings, reminding patients the aims of the study, were executed.

### Procedures

#### Training Program

The MAST sessions were held three times a week and the exercise program lasted for 10 weeks. Each exercise session consisted of a 10 min warm-up, 40 min of NW training, 20 min of strength-training exercises, and 10 min of a cool-down by stretching.

The target heart rate increased progressively from 50% up to 80%, at a 10% increase per 2 weeks, of heart rate reserve by the end of the intervention period.

Target heart rates were obtained using a combination of the HR_max_ expressed by the following formula: HR = 206 – 0.88•(age) (Gulati et al., 2010), and the Karvonen formula [(HR_max_ - HR_rest_) •(0.50 to 0.80)] + HR_rest_. The heart rate was monitored by a Polar S-610 heart-rate monitor (Polar Electro Oy, Finland).

After aerobic training, strength training (ST) was performed. ST exercises employed a subject’s own body mass and included squats, heel-raises, sit-ups, and push-ups on knees. Each exercise was performed in three sets, each set comprising the following number of repetitions: squats – 15 reps, heel-raises – 30 reps, sit-ups – up to exhaustion, push-ups on knees – 15 reps.

A fasting blood draw was completed to measure blood glucose, total cholesterol, triglycerides, and high and low-density lipoprotein cholesterol. Serum lipid levels were measured immediately on the first and last days of the training program on an empty stomach. LDL-C, HDL-C, triglycerides (TG), and total cholesterol (TC) concentrations were analyzed using the ARCHITECT ci8200 Integrated System, Abbott Diagnostic.

### Statistical Analysis

Normality of samples was tested by means of the Shapiro-Wilk test (Shapiro and Wilk, 1965) and graphically using a histogram and a quantile-quantile plot. Changes induced by the training/sedentary period in the specific parameter were analyzed by means of a t-test for repeated measures or the Wilcoxon signed-rank test. Differences between the groups were analyzed using an unpaired t-test or the Wilcoxon rank sum test.

Statistical significance was defined using the p-value of a respective statistical test. The null hypothesis of the specific test was rejected at the statistical significance level of p < 0.05. To assess the relative changes in the mean values for a specific parameter, we used the “natural” relative difference, employing natural logarithm, denoted as log percent (L%) (Tornqvist et al., 1985).

## Results

Because the analyzed data fall into parametric and non-parametric distributions, the basic statistics are represented as the median, first, and third quartile (Table 1).

There are statistically significant differences between the intervention group and the control group in lower-body strength (Training group < Control group), upper-body strength (Training group < Control group), and upper-body flexibility (Training group < Control group) at baseline (Table 1). After the training period, there was a difference between the intervention group and the control group in upper-body strength.

Application of a 10-week MAST program resulted in a statistically significant increase of VO_2max_, equal to 7.06 L%, WHR equal to 0.45 L%, lower-body strength (15.3 L%), and upper-body strength (3.09 L%). In the control group, there was a statistically significant increase in serum LDL-C levels of 0.72 L%.

In both groups, there was an improving trend of body mass and the BMI. However, in the group administered to MAST, this trend was three times greater than in the control group. There was also an improving trend in serum lipid profiles in the MAST group, i.e., a decrease in TG and LDL-C levels, and an increase in HDL-C levels.

## Discussion

The 10-week supervised MAST program improved VO_2max_, as well as the upper and lower-body strength in obese postmenopausal women. Additionally, we observed an improvement in serum TG, HDL-C, and LDL-C levels. Although, there was a significant detrimental increase in WHR, it did not fall into the cardiovascular disease risk bracket. Although, the main limitation of this study was the small sample size rendered by strict inclusion/exclusion criteria, the strength of this study was a fully supervised intervention program comprising outdoor aerobic exercises.

The results were obtained by a moderate amount (∼ 40 min) of outdoor aerobic training. Although, an earlier study indicates an improvement of 4 L% in VO_2max_ after 12-week aerobic NW training comprising three 90 min sessions (Hagner et al., 2009), application of the MAST program resulted in an improvement of ∼7 L% after only a 10-week program, comprising NW as an aerobic component of the training.

Contrary to previous results, indicating that aerobic exercises in the form of stationary cycling, at 55% of each participant’s maximal oxygen uptake, result in an acute decrease in TC levels (Lennon et al., 1983), this study did not show a decrease in the serum TC level after the MAST program. However, the MAST training led to changes in TG levels that were similar to those observed in the previous study on postmenopausal women administered to different forms of aerobic exercises (Fahlman et al., 2002; Hagner et al., 2009; Kemmler et al., 2004b).

In this study, we also showed an increase in WHR, which indicated a greater increase in the waist than this in the hip area. Since we did not study the level of fat tissue in the waist and hip areas, we were unable to assess the reasons of this phenomenon. Among possible explanation of this singularity, rendered by an analysis of Table 1, was a decrease of fat tissue in the hip area.

In the present study we showed that a combination of strength and aerobic training resulted in a significant increase of upper and lower-body strength which in turn may result in improvement of a health-related quality of life (Wiacek et al., 2009). Although, a change in VO_2max_ alone cannot serve as an indicator of a cardiac function in obese women (Lewis et al., 2010) we hypothesized that positive trends in serum lipids, an increase in HDL-C levels and a decrease in LDL-C levels, as well as VO_2max_ may serve as a predictor of an improved cardiovascular function among obese postmenopausal women administered to the MAST program.

## Conclusions

This study measured influence of a 10-week MAST program on a level of physical performance, functional fitness, anthropometry, and serum lipid levels in obese postmenopausal women. It provides insights suggesting that a MAST program, of which an aerobic component includes Nordic-walking, may be used as a means of improvement of HRQL in terms of a positive trend in serum lipid levels, VO_2max_, and body strength.

The results of this study may be further used for planning an intervention program for obese elderly women.

### Practical Implications

- A 10-week MAST program, encompassing NW as an aerobic component, increases upper and lower-body strength in obese postmenopausal women.
- A 10-week MAST program increases physical performance in terms of VO_2max_ in obese postmenopausal women.
- A 10-week MAST program results in positive changes in serum lipid levels in obese postmenopausal women.

## Figures and Tables

**Table 1:** Differences in anthropometry, serum lipids, physical performance, and functional fitness at baseline and after a 10 week (MAST) aerobic and strength training period in postmenopausal women

	Baseline (week 0)			Week 8			Log% of change		p < 0.05	

	Ex (n=23)	p	Cont (n=21)	Ex (n=23)	p	Cont (n=21)	Ex	Cont	Ex	Cont
	Median (1QR,3QR)		Median (1QR,3QR)	Median (1QR,3QR)		Median (1QR,3QR)				
*Anthropometry*										
Body mass (kg)	83.6 (75.1,91.1)		83.44 (74.9,91.1)	81.64 (73.2,90.9)		82.82 (74.2,91.1)	−1.0	−0.3		
BMI (kg/m^2^)	31.64 (28.6,34.1)		31.73 (28.5,35.1)	30.9 (27.8,33.5)		31.5 (28.3,34.2)	−1.0	−0.3		
Wc (cm)	107.75 (101.3,114.9)		109.11 (102.4,115.5)	106.79 (100.3,112.2)		109.15 (102.4,115.1)	−0.3	0.0		
Hc (cm)	111.93 (105.1,118.3)		113.65 (106.4,120.3)	110.63 (103.6,117.1)		114.56 (107.4,122.1)	−0.5	0.3		
whr	0.96 (0.9,1)		0.96 (0.95,0.97)	0.97 (0.96,0.98)		0.59 (0.58,0.6)	0.4	−0.4	*a*	
*Serum lipids*										
TC (mmol/L)	6.26 (5.6,6.7)		6.08 (5.11,7.00)	6.43 (5.7,7.2)		6.03 (5.1,7.2)	1.2	−0.4		
TG (mmol/L)	1.18 (0.9,1.4)		1.1 (0.71,1.47)	1.01 (0.7,1.2)		1.15 (0.8,1.6)	−6.8	1.9		
HDL-C (mmol/L)	1.87 (1.6,2.1)		1.93 (1.58,2.23)	2.04 (1.8,2.3)		1.87 (1.3,2.2)	3.8	−1.4		
LDL-C (mmol/L)	4.1 (3.4,4.8)		3.58 (2.65,4.51)	3.29 (2.62,3.96)		3.64 (2.8,4.5)	−1.9	0.7		*a*
*Physical performance*										
Peak VO_2max_ (ml/kg/min)	26.3 (22.2,30.1)		25.5 (22.0,28.6)	30.94 (26.6,34.3)		26 (23.1,28.5)	7.1	0.8	*a*	
*Functional fitness*										
CST (rep/sek)	0.45 (0.2,0.6)	*b*	0.68 (0.4,0.9)	0.64 (0.5,0.8)		0.63 (0.4,0.8)	15.3	−3.3	*a*	
CURL (rep/sek)	0.54 (0.5,0.7)	*b*	0.65 (0.4,0.8)	0.73 (0.5,0.8)	*b*	0.6 (0.5,0.7)	3.09	−3.5	*a*	
UBF (cm)	−5.32 (−12.9,2.4)	*b*	−6.32 (−13.0,0.5)	−5.14 (−13.0,2.5)		−6.37 (−13.0,0.4)	−1.49	0.3		
CSR (cm)	4.18 (−2.1,10.5)		1.04 (−4.2,6.4)	4.36 (−2.0,11.0)		0.99 (−4.1,6.3)	1.83	−2.1		

BMI = Body Mass Index; Ex = exercise group; Cont = control group; TC = total cholesterol; TG = triglycerides; HDL-C = high-density cholesterol; LDL-C = low-density cholesterol; SD = standard deviation

a - p-value less than 0.05 for differences in means/medians induced by the experiment;

b - p-value less than 0.05 for differences in means/medians between the experimental group and control group for the specific period of the experiment.
